# Reproductive Risk Factors Associated with Breast Cancer Molecular Subtypes among Young Women in Northern China

**DOI:** 10.1155/2020/5931529

**Published:** 2020-04-06

**Authors:** Jian Ming Wang, Jun Wang, Hong Guang Zhao, Tong Tong Liu, Fei Yang Wang

**Affiliations:** ^1^Department of Medical Imaging, Shanxi Medical University, Taiyuan, Shanxi Province, China; ^2^Department of Radiology, Shanxi Province People's Hospital, Taiyuan, Shanxi Province, China

## Abstract

**Purpose:**

Accumulated evidence suggests that reproductive factors are related to different breast cancer subtypes, but most studies on these relationships are mainly focused on middle-aged and older patients, and it remains unclear how reproductive factors impact different subtypes of breast cancer in young women.

**Methods:**

We assessed the relationships between fertility factors and luminal A, luminal B, human epidermal growth factor receptor 2 (HER2)-enriched, and triple-negative breast cancer (TNBC) subtypes in 3792 patients and 4182 controls aged 20–70 years. Data on the reproductive history of the study participants were acquired through face-to-face interviews and questionnaires. We conducted case-control comparisons among tumor subtypes based on estrogen receptor (ER), progesterone receptor (PR), and HER2 statuses using unconditional polychotomous multivariate logistic regression models to compute odds ratios (ORs) and 95% confidence intervals (CIs).

**Results:**

Parity was inversely related to both luminal A and luminal B subtypes in young women and older women (all *P*_trend_ < 0.05). Later age at first full-term birth was inversely related to the luminal A subtype (*P*_trend_ < 0.05) in young women but correlated with an increased risk of the luminal A subtype (*P*_trend_ < 0.05) in older women. Parous Chinese women 40 years old or younger who breastfed for 12 months or longer had a lower risk of luminal B and TNBC subtypes than women who never breastfed (OR = 0.55, 95% CI 0.36-0.84 and OR = 0.52, 95% CI 0.28-0.99, respectively).

**Conclusions:**

Our results implied that parity exerted a strong protective effect against luminal A and luminal B subtype breast cancer in young Chinese women, and long-term breastfeeding obviously decreased the risk of luminal B and TNBC subtypes in this population.

## 1. Introduction

Breast cancer is universally accepted as a heterogeneous disease with different molecular subtypes. Molecular subtypes of breast cancer are defined by the expression of hormone receptors and human epidermal growth factor receptor 2 (HER2). Many studies have shown that different subtypes of breast cancer have diverse clinicopathological features and prognoses [1–3]. The results of meta-analyses have suggested that fertility factors impact the etiology of breast cancer across tumor subtypes for women of diverse races in the globe [4–7]. In general, the underlying biological mechanisms remain poorly understood. To date, most studies have focused on women who were 50-60 years old and susceptible to breast cancer [8–15], whereas few studies have investigated breast cancer in young women (BCYW), especially patients 40 years old or younger. Compared with breast cancer in older patients, BCYW usually displays different molecular subtypes that have more aggressive biological characteristics. In addition, BCYW tends to be detected in an advanced stage and carry a poor prognosis, which suggests that the pathogenesis of the disease in young patients is different from that in older patients [16–18].

Several studies have shown that the proportions of luminal B and triple-negative breast cancer (TNBC) subtypes are significantly higher in young patients than in older patients [19, 20]. Six studies have investigated correlations between fertility factors and molecular subtypes of BCYW. Four of all studies focused on patients aged 20-44 years, while the rest focused on patients under 40 years of age. These studies were all carried out in Western countries, and similar studies in young women from Asian countries are lacking. Less than 7% of breast cancer cases in Western countries are diagnosed in patients 40 years old or younger [21]. The corresponding proportion is 13.2% in Asian populations [22], far higher than that in Western populations. Unfortunately, the proportion is between 15% and 20% in Chinese patients [23]. Nevertheless, the mechanism underlying how reproductive factors impact different molecular subtypes of BCYW is unclear. In this study, we analyzed the relationships between reproductive factors and molecular subtypes of breast cancer in women according to age (≤40 *vs*. >40 years).

## 2. Methods

### 2.1. Study Subjects

From Mar 2012 to Dec 2017, 3912 incident female patients without history of any previous cancer at the People's Hospital of Shanxi Medical University were confirmed by surgery and pathology as having invasive breast cancer. Ultimately, 3792 patients were enrolled in the analysis, excluding 74 for whom molecular subtype confirmation was difficult (i.e., ER-/PR+) and 46 for whom reproductive history information was missing. Controls were selected from among healthy women who had not any malignant tumor and had routine breast mammography screening during the same period. For 4312 women, the mammographic and breast ultrasound screening report was negative during the same period of time, followed by a negative mammographic and breast ultrasound examination one year later. In total, 4182 healthy women who agreed to participate in the study were randomly assigned to the control group, with matching for age (within five years), excluding 130 women who were unwilling to provide to relevant information. Our study was approved by the Ethics Review Board of the People's Hospital of Shanxi Medical University and was carried out according to the guidelines of the Declaration of Helsinki. All participants signed informed consent forms prior to their participation in this study.

### 2.2. Data Collection

All information from the participants in the study was acquired by a trained staff using face-to-face interviews and a standardized questionnaire, including reproductive risk factors and other relevant risk factors. Reproductive risk factors included age at menarche, number of full-term births, age at first full-term birth, and breastfeeding duration. Ever breastfeeding was defined as lactation for more than one month. Other relevant risk factors included age at admission, family history of breast cancer in first-degree relatives, body mass index (BMI), history of smoking and drinking, and history of oral contraceptive use. For variables with missing data (i.e., age at menarche), we supplemented these data using the average value for the variable among controls in the same age group.

### 2.3. Assessment of Tumor Biomarkers

The estrogen receptor (ER), progesterone receptor (PR), and HER2 statuses of all patients were evaluated by pathologists using rabbit monoclonal antibody for immunohistochemical (IHC) assays. We adopted high-pressure cooking for antigen retrieval. Data on all tumor biomarkers were retrieved from medical records. All invasive tumors were reevaluated by pathologists to confirm their aggressiveness. Subtypes and biomarkers of breast cancer were defined in accordance with the consensus at the 2013 St. Gallen meeting [24]. ER/PR status was considered positive when more than 1% of tumor cell nuclei were stained [25]. The assessment of HER2 status was performed according to the following IHC scores: a score of 0 or 1+ was considered negative, a score of 3+ was considered positive, and a score of 2+ required validation using fluorescent in situ hybridization (FISH) [26]. Positive results on FISH indicated that HER2 status was positive. For the Ki-67 index, tumors with ≥20% of cell nuclei stained were considered to have high proliferation. Tumor subtypes were grouped into the following categories: luminal A (ER+/PR+/HER2−, Ki − 67 < 20%), luminal B/HER2+ (ER+/HER2+/any Ki-67/any PR), luminal B/HER2− (ER+/HER2− and at least one of Ki − 67 ≥ 20% or PR−), HER2-enriched (ER−/PR−/HER2+), and TNBC (ER−/PR−/HER2−) [24]. Luminal B/HER2- and luminal B/HER2+ were regarded as a single luminal B subtype in this study.

### 2.4. Statistical Analyses

An unconditional polychotomous logistic model was used to assess the influence of fertility factors on the risk of different subtypes of breast cancer, and odds ratios (ORs) and corresponding 95% confidence intervals (CIs) were used as an assessment of the relative risk adjusted for potential confounders [27]. We considered the following factors as potential confounders in all multivariate models: age (≤25, 26-30, 31-35, 36-40, 41-45, 46-50, 51-55, 56-60, 61-65, and 66-70 years), year of recruitment (2012, 2013, 2014, 2015, 2016, and 2017), body mass index (BMI < 23, 23–24.99, 25-27.49, and ≥27.5 kg/m^2^), history of oral contraceptive use (ever/never), and family history of breast cancer (yes/no).

The number of completed pregnancies (never pregnant, 1, ≥2) was considered potential confounders when we assessed the influence of breastfeeding on the risk of different tumor subtypes. For trend analysis, categorical variables were treated as ordered discrete variables, e.g., 0, 1, and 2. The multivariate polytomous logit model was applied to assess heterogeneity between different subtypes, with luminal A breast cancer as the reference. We also examined multiplicative interaction terms generated by age at diagnosis between reproductive factors and tumor subtypes using logistic regression models (using luminal A subtype as the reference subtype) and models stratified by age (≤40/>40 years of age). We considered the analysis results to be statistically significant when *P* values were less than 0.05. SPSS version 22.0 (IBM Inc., Chicago, IL, USA) was used for all statistical analyses.

## 3. Results

The 3792 case participants comprised 3075 patients older than 40 years and 717 patients 40 years old or younger. According to age, tumor subtypes in the 3792 patients were classified as follows: luminal A (*n* = 86, 12.0%), luminal B (*n* = 420, 58.6%), HER2-enriched (*n* = 75, 10.4%), and TNBC (*n* = 136, 19.0%) in the young group and luminal A (*n* = 1080, 35.1%), luminal B (*n* = 1366, 44.4%), HER2-enriched (*n* = 316, 10.3%), and TNBC (*n* = 313, 10.2%) in the older group. Young women had a lower mean age at menarche than did older women. The mean number of live births was lower, and the mean age at first full-term birth was higher for young women than for older women. Similarly, the proportion of women who had ever breastfed was lower, and the mean duration of breastfeeding was shorter for young women than for older women. The distribution of other risk factors, including oral contraceptive use, BMI, and family history, is presented in Table 1.

As shown in Table 2, no significant correlation was found between age at menarche and the risk of any tumor subtypes in older women (all *P* > 0.05). The number of live births was not related to the risk of HER2-enriched or TNBC subtypes (both *P*_trend_ > 0.05) but was significantly inversely associated with the risk of the other two subtypes (both *P*_trend_ < 0.05). There was no significant difference between each subtype and the luminal A subtype in relation to the number of live births (all *P* > 0.05). Compared to nulliparous women, parous women had a lower risk of either the luminal A or luminal B subtype among older women, with odds ratios of 0.48 (95% CI: 0.32, 0.73) and 0.57 (95% CI: 0.38, 0.75), respectively. Moreover, among these women, two or more live births were correlated with a lower risk of the luminal A subtype compared to only one birth (OR = 0.84, 95% CI 0.73-0.97). Older age at first full-term birth was correlated only with an increased risk of the luminal A subtype in parous women older than 40 years (*P*_trend_ < 0.05). Furthermore, a significant difference existed between each subtype and the luminal A subtype regarding the age at first full-term birth in older women (all *P* < 0.05). In older women, a longer duration of breastfeeding was inversely correlated with the luminal A subtype (*P*_trend_ < 0.05) but not correlated with the other three subtypes (all *P*_trend_ > 0.05). Finally, no significant difference existed between each subtype and the luminal A subtype in terms of breastfeeding (all *P* > 0.05).

No significant correlation was found between age at menarche and the risk of any tumor subtype in young women (all *P*_trend_ > 0.05; Table 3). The number of live births was significantly correlated with a decreased risk of luminal A and luminal B subtypes (both *P*_trend_ < 0.05) in young women but was not correlated with the risk of the HER2-enriched or TNBC subtype (both *P*_trend_ > 0.05). However, relative to women who had never given birth, parous women had a lower risk of either the luminal A or luminal B subtype among young women, with odds ratios of 0.41 (95% CI: 0.19, 0.90) and 0.40 (95% CI: 0.25, 0.62), respectively. There was a significant difference between the HER2-enriched and luminal A subtypes in relation to parity in young women (*P* < 0.05). Two or more live births were not correlated with the risks of any subtype compared to only one birth among young women, while older age at first full-term birth was inversely correlated with the luminal A subtype (*P*_trend_ < 0.05). In addition, a longer breastfeeding duration was correlated with a reduced risk of luminal B and TNBC subtypes in these women (both *P*_trend_ < 0.05), but no significant correlation was observed between breastfeeding and either the luminal A or HER2-enriched subtype (both *P*_trend_ > 0.05).

Additionally, in the test of heterogeneity, there was no significant difference between each subtype and the luminal A subtype for age at first live birth and breastfeeding (all *P* > 0.05).

Subsequently, we assessed associations between parity/breastfeeding and breast cancer subtypes according to age at diagnosis (≤40 *vs.* >40 years, Supplemental Table ([Supplementary-material supplementary-material-1]). We only observed that parous women 40 years old or younger were less likely to develop HER2-enriched tumors compared to luminal A tumors (OR = 0.01, CI 0.00-0.90, *P* < 0.05), but this association did not vary significantly by age (*P* for interaction > 0.05). Compared to luminal A tumors, we did not observe a significant association between breastfeeding and luminal B and HER2-enriched subtypes across ≤40 ages and >40 ages (all *P* > 0.05). For the TNBC subtype, there was no significant association with breastfeeding across all ages (both *P* > 0.05). However, we did observe a weak but significant interaction between breastfeeding and >40 ages (*P* for interaction = 0.04).

## 4. Discussion

In this study, BCYW 40 years old or younger accounted for 18.9% of all breast cancer cases. Among young patients, the proportion of the luminal B subtype was the greatest, followed by TNBC. Furthermore, the proportion of the luminal B or TNBC subtype was higher than that in older women. This distribution of tumor subtypes for BCYW is consistent with that shown in previous studies [19, 20]. Recently, a study by Tang et al. from China found that the luminal B subtype was associated with a higher mortality and relapse rate in young patients than all subtypes in older patients [28]. However, luminal B was the most common subtype for BCYW in China, which suggests that there may be a unique etiology and biology of breast cancer in young Chinese patients [29]. In this study, the most prominent risk factors across various tumor subtypes in different age groups were parity, age at first full-term birth, and duration of breastfeeding.

A meta-analysis by Lambertini et al. showed that parity correlated with a 25% decrease in the risk of developing luminal breast cancer in parous women [7]. The hypothesized mechanism is that parity prevents breast cancer mainly by inducing changes in circulating hormones and increasing the differentiation of mammary gland tissues [30]. Similarly, we observed that parity reduced the risk of luminal A and luminal B tumor subtypes in both young and older women. Our findings in BCYW were in accordance with previous case-control studies in young women (<45 years) [31, 32]. However, we did not observe that this association differed significantly according to age (*P* > 0.05).

The results of four previous studies showed that associations between parity and the risk of TNBC were inconsistent in young parous women < 45 years. Li et al. observed that parity was associated with a reduced risk of developing TNBC in parous women < 45 years [31], whereas other studies reported the opposite [32–34], as confirmed by our results. While parity has been confirmed to be related to a decreased risk of luminal BCYW, its relationship with TNBC remains uncertain. Further studies are thus needed to confirm this association.

A prior study found parity to be a strong protector against breast cancer in women when the first full-term birth occurred between 20 and 35 years of age [35]. Another previous study showed that a later age at first full-term birth (older than 24 years) increased the risk of luminal tumor subtypes [7]. The assumption is that the pregnancy-induced differentiation of mammary tissues renders the breast less vulnerable to carcinogenic events, especially when this induced differentiation occurs at an early age [36]. We also observed that a later age at first full-term birth was related to an increased risk of luminal A breast cancer in older women. Interestingly, we found an inverse correlation between a later age at first birth and the luminal A subtype in young women, which was inconsistent with the results of prior studies on BCYW [31–34]. This discrepancy is possibly related to a different definition of later age at first full-term birth among studies. Therefore, the association between age at first-term birth and luminal A subtype for young women requires further verification.

Several meta-analyses have shown that breastfeeding exerts a protective effect against luminal and TNBC subtypes, especially TNBC, with a stronger protective effect than for other subtypes [5–7]. Two studies from China also demonstrated that long-term breastfeeding protects women against luminal and TNBC breast cancer [14, 15]. Similar to previous studies, we observed that a longer duration of breastfeeding was significantly related to a decreased risk of luminal A tumors in older patients. A study by Fabiola et al. in premenopausal Caucasian women reported that breastfeeding for more than 12 months exerted a strong protective effect against luminal B breast cancer [37]. Most notably, we found that young parous women who breastfed for 12 months or longer had a 45% lower risk of developing luminal B breast cancer than did women who never breastfed. The protection against breast cancer conferred by breastfeeding may occur through hormonal mechanisms including a reduction in estrogen levels in breast tissues, the promotion of mammary tissue differentiation, and progenitor cell apoptosis [38].

Li et al. demonstrated that breastfeeding for 12 months or longer correlates with a 50% reduction in the risk of TNBC in young parous women compared to women who never breastfed [31]. Two studies from Hui-yuan et al. on BCYW showed that in young parous women, breastfeeding for 6 months or longer correlated with a more pronounced decrease in the risk of TNBC than never breastfeeding (OR = 0.18 and 0.45, respectively) [32, 39]. We also found that young parous women who breastfed for 12 months or longer had a 48% lower risk of developing TNBC than did women who never breastfed. Longer breastfeeding durations may promote degeneration of the terminal duct lobular unit, resulting in a reduced risk of breast cancer, particularly TNBC [40, 41].

In this case-case study, we observed that parity is related to a lower possibility of being developed with HER2-enriched breast tumors compared to luminal A tumors for women ≤ 40 years, and this relationship did not differ significantly according to age (*P* > 0.05). Seemingly pregnancy produces a protective effect against HER2-enriched tumor. In parous women (>40 years), breastfeeding was not associated with TNBC tumor subtype when using luminal A tumor as the reference. However, we did observe that this association differed significantly by age (*P* = 0.04), which was related to parous women (>40 years) with TNBC tumors having different lactation ratios.

Our data provide some evidence that parity and long-term breastfeeding may be important for preventing luminal B breast cancer in young Chinese women. In general, young women in China are more susceptible to luminal B subtypes, with a poorer prognosis than all subtypes in older patients [28]. Additionally, young parous women with long-term breastfeeding have a lower risk of TNBC that is related to greater aggressiveness and a poorer prognosis. Furthermore, the results indicated that reproductive events are important in BCYW and that there may be a unique pathogenic mechanism for this group.

This study has some limitations. The first limitation of our study was the effect of memory on the collected information for the case-control study, but all questionnaires for both cases and controls were completed before diagnoses or screening. The second limitation is a potential bias in the selection of controls because healthy women who undergo breast screening may have a greater awareness of protective mechanisms against breast cancer. For example, the duration of breastfeeding in control individuals who are aware of breast cancer risk may be longer than that in their counterparts. The final limitation of our study was the insufficient number of cases due to the lower morbidity of BCYW.

In conclusion, the results of our study indicated that parity (*vs.* no parity) had a strong protective effect against luminal A and luminal B tumor subtypes in young women, and breastfeeding for 12 months or longer (*vs.* never breastfeeding) largely reduced the risk of luminal B and TNBC subtypes in young women.

## Figures and Tables

**Table 1:** Characteristics of the participants in the young and older groups.

Characteristic	Overall participants					
	Controls (*n* = 983)	≤40 years (*n* = 717)	*P* value	Controls (*n* = 3199)	>40 years (*n* = 3075)	*P* value
Mean age, years (SD, range)	35.38 (4.01, 21-40)	34.90 (4.18, 20-40)	0.121	53.36 (8.27, 41-70)	53.14 (8.37, 41-70)	0.364
Mean age at menarche, years (SD, range)	13.83 (1.39, 10-19)	13.73 (1.56, 9-19)	0.002	14.45 (1.66, 9-21)	14.41 (1.72, 10-21)	0.021
Among parous women	931 (94.7%)	645 (90.0%)	<0.001	3142 (98.2%)	2979 (96.9)	0.001
Mean number of completed pregnancies (SD)	1.44 (0.66)	1.38 (0.53)	0.001	1.70 (0.84)	1.71 (0.83)	0.005
Mean age at first completed pregnancy (SD)	25.40 (3.25)	25.46 (3.04)	0.481	24.59 (3.18)	24.71 (3.16)	0.368
Ever breastfed	865 (92.9%)	577 (89.5%)	0.016	2964 (94.3%)	2767 (92.9%)	0.020
Mean duration of breastfeeding among those who breastfed, months (SD)	16.83 (10.35)	16.45 (10.09)	0.176	25.21 (19.52)	24.76 (15.88)	<0.001
Duration of oral contraceptive use	136 (13.8%)	134 (18.7%)	0.007	458 (14.3%)	495 (16.1%)	0.049
Mean body mass index (SD)	22.87 (2.73)	23.51 (3.10)	<0.001	23.74 (2.76)	24.27 (2.61)	0.001
First-degree family history of breast cancer	40 (4.1%)	35 (4.9%)	0.421	185 (5.8%)	166 (5.4%)	0.507
Subtype of breast cancer						
Luminal A		86 (12.0)			1080 (35.1)	
Luminal B		420 (58.6)			1366 (44.4)	
HER2 enriched		75 (10.4)			316 (10.3)	
TNBC		136 (19.0)			313 (10.2)	
Annual recruitment number						
2012	129	99		485	474	
2013	145	108		516	500	
2014	176	130		534	504	
2015	165	121		525	509	
2016	172	122		557	535	
2017	196	137		582	553	

**Table 2:** Multivariate-adjusted ORs and 95% confidence intervals (CIs) for associations between fertility factors and risk of invasive breast cancer by tumor subtype in older women (>40 years of age).

Reproductive characteristics	Controls	Luminal A		Luminal B		HER2 enriched		TNBC	
	*n* (%)	*N* (%)	OR (95% CI)	*N* (%)	OR (95% CI)	*N* (%)	OR (95% CI)	*n* (%)	OR (95% CI)
Age at menarche									
≤13	1039 (32.5)	368 (34.1)	1.00 (reference)	456 (33.4)	1.00 (reference)	115 (36.4)	1.00 (reference)	104 (33.2)	1.00 (reference)
14	695 (21.7)	212 (19.6)	0.88 (0.72-1.07)	278 (20.4)	0.92 (0.77-1.10)	70 (22.2)	0.90 (0.66-1.23)	69 (22.0)	1.00 (0.73-1.38)
≥15	1465 (45.8)	500 (46.3)	0.99 (0.85-1.17)	632 (46.3)	1.01 (0.87-1.17)	131 (41.5)	0.79 (0.60-1.03)	140 (44.7)	0.96 (0.73-1.25)
*P*_trend_			0.985		0.833		0.078		0.743
*P* value for homogeneity of trends					0.854		0.206		0.899
Parity vs. no parity									
Never	57 (1.8)	38 (3.5)	1.00 (reference)	41 (3.0)	1.00 (reference)	7 (2.2)	1.00(reference)	10 (3.2)	1.00 (reference)
Ever	3142 (98.2)	1042 (96.5)	0.48 (0.31-0.73)	1325 (97.0)	0.57 (0.38-0.85)	309 (97.8)	0.77 (0.35-1.71)	303 (96.8)	0.55 (0.28-1.08)
1	1522 (47.6)	536 (49.6)	0.52 (0.34-0.79)	666 (48.8)	0.60 (0.40-0.91)	155 (49.1)	0.81 (0.36-1.81)	141 (45.0)	0.53 (0.26-1.06)
≥2	1620 (50.6)	506 (46.9)	0.44 (0.29-0.67)	659 (48.2)	0.54 (0.35-0.81)	154 (48.7)	0.73 (0.33-1.63)	162 (51.8)	0.56 (0.28-1.12)
*P*_trend_			<0.001		0.008		0.294		0.815
*P* value for homogeneity of trends					0.401		0.372		0.144
Number of live births									
1	1522 (48.4)	536 (51.4)	1.00 (reference)	666 (50.3)	1.00 (reference)	155 (50.2)	1.00 (reference)	141 (46.5)	1.00 (reference)
≥2	1620 (51.6)	506 (48.6)	0.84 (0.73-0.97)	659 (49.7)	0.89 (0.78-1.02)	154 (49.8)	0.90 (0.71-1.14)	162 (53.5)	1.07 (0.84-1.36)
Age at first live birth									
<25	1639 (52.2)	485 (46.5)	1.00 (reference)	718 (54.2)	1.00 (reference)	173 (56.0)	1.00 (reference)	159 (52.5)	1.00 (reference)
25-29	1305 (41.5)	467 (44.8)	1.24 (1.07-1.44)	514 (38.8)	0.92 (0.81-1.06)	120 (38.8)	0.88 (0.69-1.12)	129 (42.6)	1.02 (0.80-1.30)
≥30	198 (6.3)	90 (8.6)	1.58 (1.21-2.07)	93 (7.0)	1.09 (0.84-1.42)	16 (5.2)	0.77 (0.45-1.32)	15 (5.0)	0.79 (0.45-1.37)
*P*_trend_			<0.001		0.738		0.201		0.671
*P* value for homogeneity of trends					<0.001		0.002		0.021
Breastfeeding (parous women)									
Never	178 (5.7)	82 (7.9)	1.00 (reference)	94 (7.1)	1.00 (reference)	18 (5.8)	1.00 (reference)	18 (5.9)	1.00 (reference)
Ever	2964 (94.3)	960 (92.1)	0.72 (0.55-0.95)	1231 (92.9)	0.80 (0.62-1.04)	291 (94.2)	0.99 (0.60-1.64)	285 (94.1)	0.94 (0.57-1.55)
< 6 months	174 (5.5)	57 (5.5)	0.71 (0.47-1.06)	78 (5.9)	0.87 (0.60-1.25)	17 (5.5)	1.01 (0.50-2.03)	18 (5.9)	1.00 (0.50-2.00)
6–11 months	241 (7.7)	93 (8.9)	0.85 (0.60-1.22)	107 (8.1)	0.87 (0.62-1.22)	23 (7.4)	0.98 (0.51-1.87)	20 (6.6)	0.82 (0.42-1.60)
≥ 12 months	2549 (81.1)	810 (77.7)	0.70 (0.53-0.93)	1046 (78.9)	0.79 (0.61-1.02)	251 (81.2)	0.99 (0.60-1.64)	247 (81.5)	0.94 (0.57-1.56)
*P*_trend_			0.022		0.059		0.973		0.859
*P* value for homogeneity of trends					0.529		0.193		0.220

All odds ratios (OR) are adjusted for the 5-year age group, year of recruitment (2012, 2013, 2014, 2015, 2016, and 2017), oral contraceptive use (yes, no), BMI (<23, 23–24.99, 25-27.49, and ≥27.5 kg/m^2^), and family history of breast cancer (yes, no). Breastfeeding history was adjusted for the number of live births.

**Table 3:** Multivariate-adjusted ORs and 95% confidence intervals (CIs) for associations between fertility factors and risk of invasive breast cancer by tumor subtype in the younger group (≤40 years).

Reproductive characteristics	Controls	Luminal A		Luminal B		HER2 enriched		TNBC	
	*n* (%)	*N* (%)	OR (95% CI)	*N* (%)	OR (95% CI)	*N* (%)	OR (95% CI)	*n* (%)	OR (95% CI)
Age at menarche									
≤13	466 (47.4)	42 (48.8)	1.00 (reference)	204 (48.6)	1.00 (reference)	38 (50.7)	1.00 (reference)	68 (50.0)	1.00 (reference)
14	236 (24.0)	21 (24.4)	0.99 (0.57-1.71)	92 (21.9)	0.93 (0.69-1.26)	17 (22.7)	0.93 (0.51-1.69)	25 (18.4)	0.74 (0.46-1.21)
≥15	281 (28.6)	23 (26.7)	0.91 (0.54-1.56)	124 (29.5)	1.03 (0.79-1.35)	20 (26.7)	0.93 (0.53-1.64)	43 (31.6)	1.08 (0.72-1.64)
*P*_trend_			0.750		0.883		0.782		0.836
*P* value for homogeneity of trends					0.766		0.888		0.755
Parity vs. no parity									
Never	52 (5.3)	10 (11.6)	1.00 (reference)	47 (11.2)	1.00 (reference)	5 (6.7)	1.00 (reference)	10 (7.4)	1.00 (reference)
Ever	931 (94.7)	76 (88.4)	0.40 (0.18-0.88)	373 (88.8)	0.40 (0.25-0.62)	70 (93.3)	0.76 (0.27-2.11)	126 (92.6)	0.85 (0.40-1.81)
1	599 (60.9)	54 (62.8)	0.44 (0.20-0.98)	237 (56.4)	0.41 (0.26-0.65)	37 (49.3)	0.68 (0.24-1.92)	88 (64.7)	0.93 (0.43-1.99)
≥2	332 (33.8)	22 (25.6)	0.32 (0.14-0.77)	136 (32.4)	0.38 (0.23-0.61)	33 (44.0)	0.92 (0.32-2.65)	38 (27.9)	0.69 (0.31-1.55)
*P*_trend_			0.029		0.005		0.484		0.178
*P* value for homogeneity of trends					0.316		0.015		0.431
Number of live births									
1	599 (64.3)	54 (71.1)	1.00 (reference)	237 (63.5)	1.00 (reference)	37 (52.9)	1.00 (reference)	88 (69.8)	1.00 (reference)
≥2	332 (35.7)	22 (28.9)	0.73 (0.44-1.23)	136 (36.5)	0.93 (0.72-1.20)	33 (47.1)	1.40 (0.85-2.30)	38 (30.2)	0.74 (0.49-1.11)
Age at first live birth									
<25	348 (37.4)	37 (48.7)	1.00 (reference)	157 (42.1)	1.00 (reference)	28 (40.0)	1.00 (reference)	52 (41.3)	1.00 (reference)
25-29	488 (52.4)	34 (44.7)	0.65 (0.40-1.05)	183 (49.1)	0.88 (0.68-1.14)	35 (50.0)	0.99 (0.59-1.67)	63 (50.0)	0.92 (0.62-1.36)
≥30	95 (10.2)	5 (6.6)	0.48 (0.18-1.27)	33 (8.8)	0.78 (0.50-1.22)	7 (10.0)	1.05 (0.44-2.54)	11 (8.7)	0.86 (0.43-1.73)
*P*_trend_			0.043		0.209		0.949		0.598
*P* value for homogeneity of trends					0.263		0.243		0.293
Breastfeeding (parous women)									
Never	66 (7.1)	7 (9.2)	1.00 (reference)	41 (11.0)	1.00 (reference)	6 (8.6)	1.00 (reference)	14 (11.1)	1.00 (reference)
Ever	865 (92.9)	69 (90.8)	0.75 (0.33-1.70)	332 (89.0)	0.58 (0.38-0.88)	64 (91.4)	0.80 (0.33-1.94)	112 (88.9)	0.62 (0.34-1.15)
<6 months	59 (6.3)	5 (6.6)	0.75 (0.22-2.50)	30 (8.0)	0.69 (0.38-1.25)	5 (7.1)	0.83 (0.24-2.91)	15 (11.9)	1.11 (0.49-2.52)
6–11 months	141 (15.1)	13 (17.1)	0.82 (0.31-2.17)	59 (15.8)	0.67 (0.40-1.10)	12 (17.1)	1.06 (0.38-3.00)	25 (19.8)	0.84 (0.41-1.72)
≥12 months	665 (71.4)	51 (67.1)	0.73 (0.32-1.68)	243 (65.1)	0.55 (0.36-0.84)	47 (67.1)	0.75 (0.30-1.83)	72 (57.1)	0.52 (0.28-0.99)
*P*_trend_			0.492		0.005		0.428		0.004
*P* value for homogeneity of trends					0.580		0.962		0.209

All odds ratios (OR) are adjusted for 5-year age group, year of recruitment (2012, 2013, 2014, 2015, 2016, and 2017), oral contraceptive use (yes, no), BMI (<23, 23–24.99, 25-27.49, and ≥27.5 kg/m^2^), and family history of breast cancer (yes, no). Breastfeeding history was adjusted for the number of live births.

## Data Availability

The raw data is available to all reader by e-mail. There are not supplementary materials of manuscript.

## Supplementary Materials

Supplementary Materials The table showed multiplicative interaction terms generated by age at diagnosis between reproductive factors and tumor subtypes, using logistic regression models (using luminal A subtype as the reference subtype) and models stratified by age (≤40/>40 years of age).
