# Supplementary material for: Reproductive Risk Factors Associated with Breast Cancer Molecular Subtypes among Young Women in Northern China
Source: Biomed Res Int. 2020 Apr 6;2020:5931529. doi: 10.1155/2020/5931529 (PMC7166267; doi:10.1155/2020/5931529)
Supplement: Supplementary Materials — The table showed multiplicative interaction terms generated by age at diagnosis between reproductive factors and tumor subtypes, using logistic regression models (using luminal A subtype as the reference subtype) and models stratified by age (≤40/>40 years of age). [file 5931529.f1.pdf]

**Supplemental Table**							
Association between parity and breastfeeding and BC subtypes for age overall and for ages (≦40 and >40 years)							
		Parity (ever versus never)			breastfeeding (ever versus never)		
Age at breast cancer diagnosis	Molecular subtype	Odds ratio (95% CI)	P value	P value interaction parity × age	Odds ratio (95% CI)	P value	P value interaction breastfeeding × age
All age	Luminal A	1.00 (Ref.)			1.00 (Ref.)		
	Luminal B	1.72(0.72-4.12)	0.22	0.17	1.52(0.61-3.80)	0.37	0.30
	HER2 enriched	0.96(0.22-4.16)	0.95	0.61	1.07(0.24-4.70)	0.93	0.65
	TNBC	0.67(0.20-2.29)	0.52	0.62	0.78(0.63-0.97)	0.03	0.06
≤40	Luminal A	1.00 (Ref.)			1.00 (Ref.)		
	Luminal B	0.91(0.11-7.54)	0.93	0.93	0.10(0.00-4.25)	0.23	0.18
	HER2 enriched	0.01(0.00-0.90)	0.04	0.06	0.01(0.00-2.88)	0.11	0.10
	TNBC	0.26(0.02-3.29)	0.30	0.62	0.17(0.00-10.45)	0.40	0.36
>40	Luminal A	1.00 (Ref.)			1.00 (Ref.)		
	Luminal B	1.39(0.23-8.44)	0.72	0.57	1.19(0.31-4.64)	0.80	0.68
	HER2 enriched	5.02(0.17-146.82)	0.35	0.23	1.16(0.11-11.71)	0.90	0.69
	TNBC	0.20(0.01-3.98)	0.29	0.31	9.23(0.81-106.06)	0.07	0.04
All odds ratios (OR) are adjusted for 5-year age group,year of recruitment,oral contraceptive use (yes, no), BMI (<23, 23–24.99,25-27.49, ≥27.5 kg/m2) and family history of breast cancer (yes, no).							
